# Charles A. Meyer, D. V. S.

**Published:** 1887-10

**Authors:** 


### OBITUARY.

### CHARLES A. MEYER, D. V. S.

It is with deep regret that we are compelled to announce the death of Charles A. Meyer, Doctor of Veterinary Science, which occurred after a short illness August 16th, 1887, Doctor Meyer was born in New York City, February 28th, 1852. He began the study of veterinary medicine in the New York College of Veterinary Surgeons in the fall of 1877, and when the greater part of the faculty of that institution resigned, and founded the Columbia Veterinary College, entered the new institution with the entire class. Among the enthusiastic group of students who inaugurated the first year of the Columbia Veterinary College, Mr. Meyer was one of the ablest and most diligent. Not only did he master the various theoretical and practical branches of the science, but endowed with mechanical and artistic skill, laid the foundation for the anatomical museum of his *alma mater* by contributing numerous osteological and other preparations, mounted with elegance and skill. He graduated with the highest honors April 23d, 1879, and was on this occasion awarded both the Wilson gold medal for general excellence in all departments and the prize for proficiency in anatomy. After graduating, Dr. Meyer entered the practice of his profession. He soon established a lucrative practice, and his opinion was often sought by his colleagues at considerable distances from the city. His clinical insight and bluff honesty alike endeared him to his professional and personal friends. It is to be regretted that so little of his large clinical experience is registered in a published form. An article on reflex paraplegia in the horse, from his pen, is to be found in the inaugural number of this journal, and most of his subsequent writings in the *Veterinary Gazette*, of which he was one of the editors, and which was discontinued after two years. He was one of the founders of the Academy of Veterinary Science and a member of the Masonic Order. He leaves no family, his wife having preceded him in death by a year. He was taken ill with several others who drank water from a contaminated well on Staten Island, about three weeks before his death. Symptoms of typhoid fever manifested themselves, an intestinal hemorrhage occurred, and he gradually sank. His funeral was attended by a large delegation of veterinary and medical friends from this and several neighboring states. The profession has lost an ornament, his relatives a good son and brother, and his more intimate associates, a friend whose place can not be filled. *Requiescat in pace.* ***

